# Increased prevalence of carbapenem resistant Enterobacteriaceae in hospital setting due to cross-species transmission of the *bla*_NDM-1_ element and clonal spread of progenitor resistant strains

**DOI:** 10.3389/fmicb.2015.00595

**Published:** 2015-06-16

**Authors:** Xuan Wang, Gongxiang Chen, Xiaoyan Wu, Liangping Wang, Jiachang Cai, Edward W. Chan, Sheng Chen, Rong Zhang

**Affiliations:** ^1^Second Affiliated Hospital of Zhejiang University, Zhejiang UniversityHangzhou, China; ^2^Second People's Hospital of JiaxingJiaxing, China; ^3^People's Hospital of PinghuJiaxing, China; ^4^Shenzhen Key lab for Food Biological Safety Control, Food Safety and Technology Research Center, Hong Kong PolyU Shen Zhen Research InstituteShenzhen, China; ^5^State Key Lab of Chirosciences, Department of Applied Biology and Chemical Technology, The Hong Kong Polytechnic UniversityKowloon, Hong Kong

**Keywords:** carbapenem-resistant *Enterobacteriaceae*, NDM-1, clonal spread, mobile element

## Abstract

This study investigated the transmission characteristics of carbapenem-resistant *Enterobacteriaceae* (CRE) strains collected from a hospital setting in China, in which consistent emergence of CRE strains were observable during the period of May 2013 to February 2014. Among the 45 CRE isolates tested, 21 (47%) strains were found to harbor the *bla*_NDM-1_ element, and the rest of 24 CRE strains were all positive for *bla*_KPC-2_. The 21 *bla*_NDM-1_—borne strains were found to comprise multiple *Enterobacteriaceae* species including nine *Enterobacter cloacae*, three *Escherichia coli*, three *Citrobacter freundii*, two *Klebsiella pneumoniae*, two *Klebsiella oxytoca*, and two *Morganella morganii* strains, indicating that cross-species transmission of *bla*_NDM-1_ is a common event. Genetic analyses by PFGE and MLST showed that, with the exception of *E. coli* and *E. cloacae*, strains belonging to the same species were often genetically unrelated. In addition to *bla*_NDM-1_, several CRE strains were also found to harbor the *bla*_KPC-2_, *bla*_VIM-1_, and *bla*_IMP-4_ elements. Conjugations experiments confirmed that the majority of carbapenem resistance determinants were transferable. Taken together, our findings suggest that transmission of mobile resistance elements among members of *Enterobacteriaceae* and clonal spread of CRE strains may contribute synergistically to a rapid increase in the population of CRE in clinical settings, prompting a need to implement more rigorous infection control measures to arrest such vicious transmission cycle in CRE-prevalent areas.

## Introduction

β-Lactams have been a cornerstone in treatment of infections caused by Gram-negative bacterial pathogens due to their high efficacy and low toxicity to humans, among which carbapenems are considered agents of the last resort, especially in cases where extended-spectrum β-Lactamase (ESBL) producing organisms were involved (Dalhoff and Thomson, 2003). In the past two decades, usage of carbapenems such as imipenem and meropenem has been substantially increased due to the emergence of multidrug-resistant organisms (Goel et al., 2011; Zilberberg and Shorr, 2013). However, increased carbapenem consumption in turn initiated a vicious cycle in which carbapenem-resistant Gram-negative pathogens (CRGNP), which often cause untreatable hospital infections (Livermore, 2004, 2009; Karaiskos and Giamarellou, 2014), further gained selection advantage. Species belonging to the family *Enterobacteriaceae* are common human pathogens which can cause a wide range of community-acquired and nosocomial infections (Stock, 2014). The emergence of carbapenem-resistant *Enterobacteriaceae* (CRE) has posed a huge challenge to clinical infection control. Carbapenem resistance in CRE was mainly mediated by the production of carbapenemases, among which KPC, Metallo-β-lactamases (VIM, IMP, NDM) and OXA-48 type of enzymes were the most common (Nordmann et al., 2011). New Delhi Metallo-β-lactamase-1 (NDM-1) was one of the most important carbapenemases of CRE. Since its first discovery in 2008 in a *Klebsiella pneumoniae* isolate recovered from a patient at a hospital in New Delhi, India, it has been transmitted to many species of *Enterobacteriaceae* in various countries (Yong et al., 2009). NDM-1 is most frequently identified in the Indian subcontinent, followed by the Balkans region and the Middle East, and is mainly associated with community-acquired infections.

In China, the first clinical report of *bla*_NDM−1_ involved carbapenem-resistant *Acinetobacter baumannii* strains detectable in four patients indifferent provinces in 2011 (Chen et al., 2011). Since then it has been recoverable in most species of *Enterobacteriaceae*, including *K. pneumoniae, Klebsiella oxytoca*, *Escherichia coli*, *Enterobacter cloacae, Enterobacter aerogenes*, and *Citrobacter freundii*. To date, NDM-1-producing isolates have been reported in various cities in China including Beijing, Changsha, Chongqing, Fuzhou, Guangzhou, Hangzhou, Hebei, Hong Kong, and Zhengzhou (Berrazeg et al., 2014; Qin et al., 2014). Although various *bla*_NDM−1_-carrying CRE strains have been sporadically identified, few outbreaks of CRE carrying the *bla*_NDM−1_ element have been reported in China, suggesting that the transmission of NDM-1 is mainly mediated by conjugative plasmids, which is consistent with the features of NDM-1 transmission observable in other parts of the world (Hu et al., 2014). In this study, we report an increasing prevalence of *bla*_NDM−1_-positive *Enterobacteriaceae* in a Jiaxing hospital, which is located in Zhejiang Province, China. Through time-series analysis of the molecular features and epidemiological linkage of CRE recovered within the hospital, we demonstrated that emergence of new CRE strains was due to a combination of clonal spread of existing *bla*_NDM−1_-carrying strains and efficient horizontal transfer of the *bla*_NDM−1_ elements from such strains to other drug sensitive organisms. This combinatorial mode of transmission of both CRE organisms and the resistance elements that they harbor can theoretically result in an exponentially increasing rate of spread of NDM positive CRE in clinical settings if proper infection control measures are not implemented to disrupt the transmission routes.

## Materials and methods

### Bacterial strains and species identification

From May 2013 to February 2014, a total of 6598 clinical *Enteroabcteriaceae* strains were isolated from different specimens (urine, feces, and sputum) collected from patients in Second People's Hospital of Jiaxingin Zhejiang Province, China. All isolates were identified using the Vitek 2 system (bioMérieux, Marcy-l'E' toile, France), and confirmed by the MALDI-TOF MS apparatus (Bruker Microflex LT, Bruker Daltonik GmbH, Bremen, Germany). These isolates were screened for their ability to produce carbapenemases by a disc diffusion test, in which 10-mg imipenem discs were used (Oxoid, Basingstoke, UK) (Zhou et al., 2015). A total of 45 CRE isolates were recovered from these *Enterobacteriaceae* strains.

### Molecular detection of resistance genes

PCR and nucleotide sequencing were employed to screen for the presence of carbapenemase-encoding genes, including *bla*_VIM_, *bla*_IMP_, *bla*_KPC_, *bla*_OXA−48_ and *bla*_NDM−1_, as well as ESBL genes, including *bla*_CTX−M_, *bla*_TEM_ and *bla*_SHV_, as described previously (Dallenne et al., 2010). An imipenem-EDTA double-disc synergy test and a modified Hodge test were used to screen for the presence of Metallo-β-lactamases(MBLs) and carbapenemases, respectively, and were analyzed according to CLSI guidelines (Zhou et al., 2015).

### Antimicrobial susceptibility testing

The MICs of 10 antibiotics, including imipenem, meropenem, ceftazidime, cefotaxime, aztreonam, piperacillin-tazobactam, fosfomycin, amoxicillin-clavulanic acid, amikacin, tigecycline, were determined using the agar dilution method, and the results were analyzed according to the CLSI criteria of 2014 (Zhou et al., 2015). The 2014 EUCAST breakpoints were used (available at http://www.eucast.org/clinical_breakpoints/) for tigecycline.

### PFGE and MLST

Clonal relationships between *bla*_NDM−1_-positive isolates were investigated by PFGE of *Xba*I-digested genomic DNA using a Rotaphor System 6.0 instrument (Whatman Biometra, Goettingen, Germany), with a running time of 24 h and pulse times of 3–40 s. *Salmonella* strain H9812 was used as the control strain. Bands were stained with ethidium bromide (0.5 μg/mL) prior to visualization under UV light. A dendrogram depicting the genetic relatedness of the test strains was generated from the homology matrix with a 0.2% coefficient by the unweighted pair-group method, and by using arithmetic averages (UPGMA), to describe the relationships of the PFGE profiles. Isolates were allocated to the same PFGE group if their dice similarity index was ≥85%.

MLST was performed using seven housekeeping genes in*bla*_NDM−1_-producing *E. coli*, *K. pneumonia* and *E. cloacae* isolates, which were amplified using primers listed in the online databases (http://pubmlst.org/ecloacae/ for *E. cloacae*, http://bigsdb.web.pasteur.fr/klebsiella/klebsiella.html for *K. pneumoniae* and http://mlst.warwick.ac.uk/mlst/dbs/Ecoli for *E. coli*). The resultant PCR products were purified and sequenced. Sequence types (STs) were assigned using online database tools.

### Conjugation experiments

The conjugation experiment was carried out using the mixed broth method as previously described (Borgia et al., 2012). Both the donor (*bla*_NDM−1_-positive *Enterobacteriaceae*) and the recipient strains (sodium azide-resistant *E. coli* J53) were mixed on Luria-Bertani agar at a ratio of 1:1, and the mixtures were incubated for 24 h at 35°C. Transconjugants were selected on LB agar supplemented with sodium azide (100 mg/L) and meropenem (0.3 mg/L). Colonies that grew on the selective medium were picked for identification by the Vitek MS system. Transformants that harbored *bla*_NDM−1_ and exhibited resistance to carbapenems and cephalosporins were defined as transconjugants.

## Results

The study period and venue were May 2013 to February 2014 and the Second Hospital of Jiaxing, Zhejian Province, China, respectively. During this period, a total of 45 CRE isolates were recovered from 6598 clinical specimens (urine, feces and sputum), among which 21 (47%) were found to harbor the *bla*_NDM−1_ elements; the rest of 24 CRE strains were all positive for *bla*_KPC−2_. The objective of the study was to investigate the molecular events that lead to emergence of CRE in the hospital, with a focus on understanding the molecular and epidemiological features of transmission of both *bla*_NDM−1_-borne strains and the *bla*_NDM−1_ element itself. The 21*bla*_NDM−1−_borne CRE strains were found to comprise a variety of different species of *Enterobacteriaceae* including nine *E. cloacae*, three *E. coli*, three *C. freundii*, two *K. pneumoniae*, two *K. oxytoca* and two *M. morganii* strains, indicating that *bla*_NDM−1_can be efficiently acquired by various *Enterobacteriaceae* species. Most of these *bla*_NDM−1_–borne CRE were recovered from patients in the Neurosurgery Department (43%), who were subjected to various neuronal system surgical procedures, followed by patients from the Respiratory Department (19%) suffering mainly from pulmonary infection, patients from the Pediatric Department and Recovery department (14% each), and one patient each from the Cardio-Thoracic Surgery and Breast Department (Table 1). These *bla*_NDM−1_ borne CRE strains were isolated from urine (85%), sputum (10%), and breast secretion (5%), respectively.

**Table 1:** **Origin and molecular features of** ***Enterobacteriaceae*** **isolates harboring** ***bla*****_NDM−1_, and clinical outcome of diseases that they caused**.

**Isolate**	**Isolation date**	**Location of acquisition**	**Culture type**	**Diseases**	**Treatment**	**Outcome**	**PFGE**	**MLST**
EC-06	26/9/2013	PD	Urine	Neonatal pneumonia	Ceftazidime	Recovered	Ec1	ST167
EC-33	18/01/2014	ECD	Urine	Myelitis	NT	Uracratia	Ec2	ST167
EC-34	12/02/2014	RCD	Urine	Myelitis, Urinary tract infection	NR	NR	Ec2	ST167
KO-03	24/06/2013	ND	Urine	Subarachnoid hemorrhage, Oculomotor paralysis, Pneumonia	NR	Recovered	Ko1	ND
KO-16	15/08/2013	ND	Urine	Cerebroma recurrence	NT	Discharged	Ko1	ND
CF-05	26/06/2013	ND	Urine	Traumatic subarachnoid hemorrhage	Levofloxacin, Amikacin	Recovered	Cf1	ND
CF-17	20/08/2013	RD	Sputum	Pulmonary infection	Tigecycline	Recovered	Cf2	ND
CF-35	20/02/2013	ND	Urine	Cerebral contusion	Levofloxacin	Recovered	Cf3	ND
ECL-07	29/06/2013	PD	Urine	Neonatal pneumonia	Ceftazidime	Recovered	Ecl1	ST114
ECL-08	05/08/2013	ND	Urine	Cerebral hemorrhage	Levofloxacin	Transferred	Ecl1	ST114
ECL-09	07/08/2013	ND	Urine	Intracranial aneurysm	Piperacillin/Tazobactam	Discharged	Ecl2a	ST93
ECL-10	09/08/2013	ND	Urine	Traumatic subdural hemorrhage	Levofloxacin	Recovered	Ecl1	ST114
ECL-18	28/08/2013	PD	Urine	Neonatal infections	Ceftazidime	Recovered	Ecl3	ST190
ECL-19	28/08/2013	RD	Urine	Pulmonary infection	Mupirocin ointment		Ecl1	ST114
ECL-22	02/10/2013	ND	Urine	Subarachnoid hemorrhage	Levofloxacin	Recovered	Ecl1	ST114
ECL-27	11/11/2013	CTS	Sputum	Abdominal aortic aneurysm	Cefuroxime	Recovered	Ecl4	ST66
ECL-28	27/11/2013	RD	Urine	Chronic obstructive bronchitis	Cefoperazone/Sulbactam		Ecl2b	ST93
KP-04	26/06/2013	RD	Urine	Bladder neoplasmpulmonary infection	Ceftazidime, Piperacillin/Tazobactam	Discharged	Kp1	ST147
KP-14	08/08/2013	ND	Urine	Cerebral hernia	Levofloxacin	Discharged	Kp2	ST1724
MM-23	04/10/2013	BD	Secretion	Cholangitis	Amikacin, Metronidazole	Recovered	Mm1a	ND
MM-26	29/10/2013	RCD	Urine	Spinal cord injury	Amikacin	Recovered	Mm1b	ND

*EC, Escherichia coli; KO, Klebsiella oxytoca; CF, Citrobacter freundii; ECL, Enterobacter cloacae; KP, Klebsiella pneumoniae; MM, Morganella morganii. JH, the Second People's Hospital of JiaXing; NT, not treated; NR, no record; ND, not determined. PD, Pediatric Department; RCD, Recovery Department; ND, Neurosurgery Department; CTS, Cardio-Thoracic Surgery; RD, Respiratory Department; BD, Breast Department*.

Clinical records showed that most of patients from whom *bla*_NDM−1_–borne CRE were recovered have been subjected to different types of antimicrobial treatment including the use of cephalosporins (ceftazidime, cefoperazone/sulbactam, cefuroxime, piperacillin/tazobactam), amikacin, levofloxacin and tigecycline, and the vast majority of patients recovered and were discharged from the hospital. Our record also showed that although *bla*_NDM−1_-borne CRE were resistant to all cephalosporins, clinical treatment with cephalosporins for non-blood infections caused by *bla*_NDM−1_borne CRE remained effective (Table 1). In addition, levofloxacin could be a choice for treatment of infections caused by fluoroquinolone-susceptible CREs. Likewise, amikacin was also a good choice since most of the CRE were susceptible to this antibiotic.

**Figure 1:**
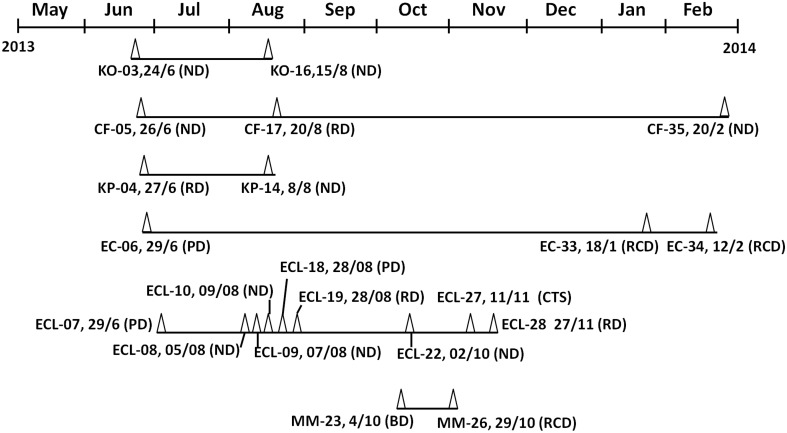
**Timeline of events in which epidemiologically linked NDM-1–producing** ***Enterobacteriaceae*** **strains were recovered**. Test strains were isolated from May2013 to February 2014 in the Second People's Hospital of Jiaxing. Dates are shown as date/month. Abbreviations: Δ, NDM-1 positive isolate; EC, *Escherichia coli*; KO, *Klebsiella oxytoca*; CF, *Citrobacter freundii*; ECL, *Enterobacter cloacae*; KP, *Klebsiella pneumoniae* MM, *Morganella morganii*. PD, Pediatric Department; RCD, Recovery Department; ND, Neurosurgery Department; CTS, Cardio-Thoracic Surgery; RD, Respiratory Department; BD, Breast Department.

The first case of CRE infection occurred in the Neurosurgery Department on June 24, 2013, with the causative organism being identified as *K. oxytoca*, recurrent infection due to the same clone with identical PFGE pattern occurred on August 15, 2013, suggesting the long-term persistence of similar clones in a hospital. Interestingly, non-clonal spread was seen in specific species of CRE such as *C. freundii* and *K. pneumoniae*. For instance, the first case of NDM-producing *C. freundii* infection was recorded on June 26, 2014 in the Neurosurgery Department, followed by the second case in the Respiratory Department on August 17, 2013; the third case was recorded back in Neurosurgery Department on February 20, 2014, however, pneumonia-associated *C. freundii* strains recoverable form each of these three cases were found to be genetically non-identical (Figure 1, Table 1). Likewise, two genetically un-related strains of *K. pneumoniae* were found to cause infections in different departments at different dates (Figure 1, Table 1). On the other hand, both non-clonal and clonal spread could be seen upon analysis of infection caused by *E. coli* and *E. cloacae*. The first case of NDM-producing *E. coli* was observable in the Pediatric Department on September 26, 2013, whereas a different strain was found to cause infections in the Recovery Department in 2014. Although the NDM-1 producing *E. coli* strains recovered belonged to two different clones, three of the strains were found to belong to ST167 (Figure 1, Table 1). *E. cloacae* were the most common CRE species in this hospital. The first case of *E. cloacae* was reported on June 29, 2013 in the Pediatric Department. The same clone, which belonged to ST114, was causing an outbreak in the Neurosurgery Department, in which four infections recorded during the period of August to October, 2013. In August 2013, infections caused by a different clone also occurred in the Pediatric Department, and other clones were also found to cause infections in different departments at different times. These clones belonged to different ST types such as ST93, ST190, and ST66 (Figure 1, Table 1). Finally, NDM-1 producing *Morganella morganii* was reported for the first time in this hospital. Two very similar clones were found to cause infections in different departments and dates (Figure 1, Table 1).

Antimicrobial susceptibilities were performed on all CRE strains. All 21*bla*_NDM−1_-positive isolates were resistant to most β-lactam antibiotics, including expanded-spectrum cephalosporins and the carbapenems. Moreover, all isolates were resistant to fosfomycin (≥256 mg/L) and amoxicillin-clavulanic acid (Table 2). In addition, 10 isolates were resistant to amikacin (≥64 mg/L). Only two isolates were sensitive to aztreonam (≤4 mg/L). Sixteen isolates exhibited intermediate susceptibility to tigecycline (MICs ≤ 1 mg/L), and 11 were tigecycline-resistant (MICs > 2 mg/L). The high frequency of resistance observed to alternative therapeutic antibiotics is a great concern for clinicians in charge of treating infections caused by these CREs.

**Table 2:** **Profiles of antimicrobial drug susceptibility and detectable-lactamase determinants of NDM-1 positive CRE strains and the corresponding transconjugants**.

**Strains**	**MICs (mg/L)**												**Additional β lactamase determinants**
	**IPM**	**MEM**	**CAZ**	**CTX**	**ATM**	**TZP**	**SCF**	**AMC**	**LEV**	**FOS**	**AK**	**TIG**	
EC-06	16	32	>256	>256	>256	>256	>256	128	8	32	4	2	CTX-M-3, CTX-M-14,SHV-12
T-EC06	16	32	>256	>256	2	>256	>256	128	<0.125		1	1	–
EC-33	4	4	>256	>256	>256	>256	>256	64	32	32	2	1	CTX-M-15
T-EC33	4	4	>256	>256	>256	>256	>256	64	<0.125		1	1	CTX-M-15
EC-34	4	8	>256	>256	>256	>256	>256	128	32	32	2	2	CTX-M-15, TEM-1,SHV-12
T-EC34	4	4	>256	>256	>256	>256	>256	64	<0.125		1	1	TEM-1,SHV-12
KO-03	8	16	>256	>256	>256	>256	>256	128	16	16	1	4	SHV-12
T-KO03	16	32	>256	>256	2	>256	>256	128	<0.125		1	1	–
KO-16	8	16	256	>256	>256	>256	>256	128	16	16	1	4	SHV-12
T-KO16	16	32	>256	>256	>256	>256	>256	128	<0.125		1	1	SHV-12
CF-05	16	32	>256	>256	>256	>256	>256	256	4	8	1	2	SHV-12
T-CF05	16	32	>256	>256	>256	>256	>256	128	<0.125		1	1	SHV-12
CF-17	8	8	>256	>256	256	>256	>256	256	8	32	16	2	SHV-12
CF-35	8	32	>256	>256	>256	>256	>256	>256	8	16	>64	1	CTX-M-3, KPC-2,SHV-12
T-CF35	2	1	16	8	128	128	32	128	<0.125	0.25	>64	0.5	SHV-12
ECL-07	4	16	>256	>256	>256	>256	>256	256	16	64	>64	2	CTX-M-14, TEM-1,SHV-12
T-ECL07	4	2	>256	128	64	256	128	256	<0.125	0.5	1	1	TEM-1
ECL-08	4	4	>256	>256	>256	>256	>256	128	16	64	>64	4	CTX-M-14,TEM-1,SHV-12
ECL-09	4	16	>256	>256	>256	>256	>256	256	32	32	>64	4	CTX-M-3, CTX-M-14, TEM-1,SHV-12
T-ECL09	4	1	>256	256	32	256	256	256	0.25	0.5	1	1	CTX-M-14,TEM-1
ECL-10	8	16	>256	>256	>256	>256	>256	256	16	>64	>64	4	CTX-M-14, TEM-1,SHV-12
T-ECL10	8	8	>256	128	128	256	256	256	<0.125	<0.125	1	1	–
ECL-18	4	32	256	>256	256	256	>256	256	0.5	1	>64	2	IMP-4,TEM-1
T-ECL18	4	8	>256	128	16	256	256	256	<0.125	<0.125	>64	1	TEM-1
ECL-19	8	8	>256	>256	>256	>256	>256	256	16	64	>64	2	CTX-M-14,KPC-2
T-ECL19	8	4	>256	128	64	256	256	256	<0.125	<0.125	1	1	–
ECL-22	8	4	>256	>256	>256	>256	>256	256	32	32	>64	4	CTX-M-14, TEM-1,SHV-12
T-ECL22	8	8	>256	128	128	>256	256	256	0.25	0.5	1	1	TEM-1,SHV-12
ECL-27	4	4	256	>256	256	>256	>256	128	0.5	2	0.5	2	CTX-M-3, CTX-M-14, TEM-1,SHV-12
T-ECL27	8	8	>256	128	128	>256	256	256	<0.125	0.5	1	1	TEM-1
ECL-28	4	4	>256	>256	256	>256	>256	256	64	>64	4	4	TEM-1,SHV-12
T-ECL28	4	8	>256	>256	256	>256	256	256	0.25	1	2	1	TEM-1,SHV-12
KP-04	8	16	>256	>256	>256	>256	>256	128	2	4	1	2	CTX-M-14, VIM-1,TEM-1,SHV-12
T-KP04	8	16	>256	>256	>256	>256	>256	128	<0.125	0.5	1	1	SHV-12
KP-14	16	16	256	>256	>256	>256	>256	128	0.15	0.5	1	2	CTX-M-14, KPC-2,SHV-12
T-KP14	16	16	256	>256	>256	>256	>256	128	0.15	0.5	1	2	SHV-12
MM-23	8	4	128	>256	2	256	>256	>256	1	4	4	2	–
T-MM23	4	4	>256	128	1	>256	256	256	2	4	1	2	–
MM-26	8	4	64	>256	2	64	>256	>256	16	16	4	4	–
T-MM26	4	8	>256	128	1	>256	256	128	1	2	1	2	–

*T denotes transconjugant. IPM, imipenem; MEM, meropenem; CAZ, ceftazidime; CTX, cefotaxime; ATM, aztreonam; TZP, piperacillin-tazobactam; CPS, cefoperazone/sulbactam; AMC, amoxicillin-clavulanic acid; LEV, levofloxacin, FOS, fosfomycin; AK, amikacin; TIG, tigecycline*.

Transferability of resistance determinants in these CRE strains, in particular those producing NDM-1, was investigated. Conjugation experiments were successful for all isolates except for two (ECL-8 and CF-17) *bla*_NDM−1_-positive isolates. The resulting 19 transconjugants all exhibited resistance to carbapenems and cephalosporins. Importantly, fosfomycin and amikacin resistance determinants could also be transferred to the recipient *E. coli* strain (Table 2). Phenotypic resistance to all β-lactams in these isolates suggested that they might express additional β-lactamases since NDM-1 would not mediate resistance to aztreonam. In view of the discrepancy between the existence of known carbapenemase genes and the drug susceptibility profiles of the test strains, all CRE strains and their corresponding transconjugants were screened for the presence of additional β-lactamase genes. Our data revealed that in addition to *bla*_NDM−1_, strains CF35, ECL19, and KP14 also harbored *bla*_KPC−2_, strain KP04 was found to harbor *bla*_VIM−1_, and *bla*_IMP−4_ was detectable in ECL18. To the best of our knowledge, this is the first case of *K. pneumoniae* containing both *bla*_NDM−1_ and *bla*_VIM−1_, hence further works are required to elucidate the mechanisms governing the uptake of multiple carbapenemase genes in a single organism. In addition, 18 out of the 21 (86%) *bla*_NDM−1_-positive isolates were found to harbor ESBL genes in various combinations (Table 2). It should also be noted that the additional resistance genes detectable in several isolates, including *bla*_SHV−12_, *bla*_CTX−M−3/14_, *bla*_TEM−1_ and *bla*_KPC−2_, could be co-transferred to the *E. coli* recipient strain J53, along with the *bla*_NDM−1_ element (Table 2).

## Discussion

Since the first report of their emergence, NDM-1-producing *Enterobacteriaceae* have become a worldwide public health concern (Patel and Bonomo, 2013). In China, currently available data tend to suggest that *bla*_NDM−1_ is only present at a relatively low frequency and spreading sporadically amongst *Enterobacteriaceae* (Wang et al., 2013; Hu et al., 2014). A recent study reported a high rate [33.3% (16/48)] of NDM-1 positive CRE organisms in a hospital in Henan Province, China, most of which were due to plasmid mediated transmission of the *bla*_NDM−1_ elements among different members of *Enterobacteriaceae* (Qin et al., 2014). Although the prevalence of *bla*_NDM−1_-positive CRE has been increasing over the past several years in China, very few epidemiological data are available to elucidate the underlying mechanisms that mediate the increased rate of transmission of NDM-1 positive CRE strains in hospitals. In this study, we identified 21 NDM-1-producing strains of *Enterobacteriaceae* in a hospital over a short period of time, from May 2013 to February 2014, with the majority of the strains (18/21) being collected during the period of June–November 2013. Our data suggest that a combination of outbreak of CRE infections and sporadic emergence of genetically unrelated resistant organisms contributed to the dramatic increase of CRE infections in this hospital during this period, prompting a need to investigate the molecular basis of these events. It should be noted that the series of CRE infections investigated in this study represents the most serious of its kind in China to date (Berrazeg et al., 2014; Zhou et al., 2014).

MLST-diverse NDM-1-producing *E. coli* (ST410, ST131, ST684, and ST101) and *K. pneumoniae* strains (ST147, ST14, ST11, and ST340) have been identified worldwide (Poirel et al., 2011). In this work, the NDM-1 positive *E. coli* strains tested were found to belong to ST167 with two distinct PFGE types. NDM-1 positive ST167 *E. coli* were previously shown to be an animal-associated clone recoverable in both France and China (Cuzon et al., 2013; Zhang et al., 2013; Yang et al., 2014). Repeated emergence of these NDM-1 positive *E. coli* strains in China represents a significant clinical and public health concern. The two *K. pneumoniae* isolates reported in the current study belonged to ST147 and a novel type, ST1724. Clinical NDM-1-producing *K. pneumoniae* ST147 strains are frequently detected worldwide, suggesting that they play an important role in the dissemination of the *bla*_NDM−1_ elements to other *K. pneumoniae* strains (Giske et al., 2012; Peirano et al., 2014). Identification of carbapenem-resistant *K. pneumoniae* strains belonging to the novel STs (ST359 and ST1724) in this study infers that the size of the existing pool of NDM-1-producing strains has been further expanded.

In contrast to *E. coli* and *K. pneumoniae*, MLST type of NDM-1-producing *E. cloacae* strains in this study included both epidemic ST types and new ST types. A carbapenem-resistant *E. cloacae* strain belonging to ST89 and producing the OXA-48 carbapenemase was isolated in Poland in 2014 (Majewski et al., 2014). Izdebski et al. reported that the ST66, ST78, and ST114 types have spread worldwide and were commonly associated with production of ESBLs and carbapenemases in many countries (Izdebski et al., 2014). NDM positive *E. cloacae* strains are among the most frequently reported CRE species and are known to cause occasional hospital outbreaks and community-acquired infections (Rozales et al., 2014; Yanik et al., 2014; Stoesser et al., 2015). In this study, we reported a similar situation in which *E. cloacae* belonging to the ST114 type was linked to five infection cases, organisms belonging to the ST93 type were linked to two cases of infections, and two new ST types, ST66 and ST190 were responsible for one infection each. Since the five ST114 isolates were collected from different wards at the Second People's Hospital of Jiaxing, we speculated that NDM-1-producing *E. cloacae* strain ST114 may have already spread in the hospital.

In the United Kingdom and the Indian subcontinent, *bla*_NDM−1_ has been found to be located in plasmids of various sizes (ca. 50–300 kb), which generally belong to at least four different Inc groups, including A/C,L/M, FI/FII and an undefined type (Kumarasamy et al., 2010; Poirel et al., 2011). In our study, most of *bla*_NDM−1_ elements recoverable from CRE isolates were harbored by self-transmissible plasmids, which also encoded multiple β-lactamases and other determinants of amikacin and fosfomycin resistance. This finding is consistent with currently available data in China and various other countries. Finally, our finding that transmission of conjugative plasmids encoding various carbapenemases and clonal spread of strains containing such plasmids were both responsible for a significant increase in the number of NDM-1 positive CRE infections in a hospital in China raised an alarming possibility that the prevalence of CRE can increase rapidly in a hospital setting within a short period. Whether the rate of infections due to such strains increases at a similar rate in the future depends on numerous factors including the effectiveness of infection control measures of the hospital concerned and the immune status of the infected patients. More works are urgently needed to investigate factors that determine the rate of transmission of CRE and the mobile resistance elements in order to help design appropriate intervention strategies that pinpoint the core of the problem, that is, to target the pool of existing CRE and the resistance elements that they harbor.

## Funding

This work was supported by the Chinese National Key Basic Research and Development Program (2013CB127200) and Natural Science Foundation of China (Grant No. 81371871).

### Conflict of interest statement

The authors declare that the research was conducted in the absence of any commercial or financial relationships that could be construed as a potential conflict of interest.
